# Effect of FLASH proton therapy on primary bronchial epithelial cell organoids

**DOI:** 10.1016/j.ctro.2025.100927

**Published:** 2025-01-29

**Authors:** Merian E. Kuipers, Floriane van Liefferinge, Ernst van der Wal, Marta Rovituso, Annelies M. Slats, Pieter S. Hiemstra, Krista C.J. Van Doorn-Wink

**Affiliations:** a Leiden University Medical Center (LUMC), Department of Pulmonology, C02-Q, Albinusdreef 2 2333 ZA Leiden, the Netherlands; b Holland Proton Therapy Center (HollandPTC), Huismansingel 4 2629 JH Delft, the Netherlands; c Leiden University Medical Center (LUMC), Department of Radiotherapy, K01-P, Albinusdreef 2 2333 ZA Leiden, the Netherlands

**Keywords:** FLASH effect, Organoid model, NSCLC, Proton radiation, Ultra-high dose rate (UHDR), Primary cells, 3D model

## Abstract

**Purpose:**

The effects of conventional (CONV) and FLASH proton therapy on primary bronchial epithelial cell (PBEC) organoids from individuals with chronic obstructive pulmonary disease (COPD) were investigated. The primary objective was to compare the effect of FLASH and CONV on COPD PBEC organoids with a focus on DNA damage, organoid formation, and gene expression.

**Methods:**

PBECs were obtained from six COPD donors, cultured as three-dimensional (3D) organoids and exposed to 2 and 8 Gy CONV and FLASH proton radiation at the Holland Proton Therapy Center. DNA damage was assessed by γH2AX staining. Organoid formation capacity was assessed by counting the organoids formed after reseeding irradiated cells at 24 h and 7 days. Bulk RNA sequencing (RNAseq) and qPCR analyses were performed to identify pathways and differences in the radiation response.

**Results:**

γH2AX foci analysis showed a significant dose-dependent increase in DNA damage at 1 h for both CONV and FLASH treatments, without differences between the two modalities. Organoid formation assays revealed a dose-dependent decrease in organoid formation capacity at 24 h for both treatments. At 7 days, 2 Gy FLASH-treated samples showed significantly reduced organoid formation compared to 2 Gy CONV (p = 0.008). RNAseq identified CONV and FLASH-induced changes in expression of DNA-damage response and apoptosis pathway genes. A dose-dependent upregulation of MDM2, GDF15, DDB2, BAX, P21, AEN and a decrease in MKi67 expression was confirmed by qPCR analysis.

**Conclusion:**

No significant differences were found in DNA damage or gene expression profiles between CONV and FLASH. The organoid formation assay showed a prolonged detrimental effect in the FLASH-treated organoids, suggesting a more complex interaction of FLASH with lung epithelial cells. The results of this study contribute to the advancement of robust *in vitro* human lung models for investigating the mechanisms of action of FLASH, potentially facilitating the treatment of NSCLC patients with proton FLASH therapy.

## Highlights

• This is the first study with primary 3D lung organoids investigating proton FLASH.
• Protons decrease the progenitor function in human lung epithelial organoids.
• No sparing effect was observed in organoids irradiated with conventional or FLASH protons.
• More complex models with alveolar, endothelial and immune cells could provide further insight.

## 1 Introduction

Radiotherapy is an essential part of the treatment for non-small cell lung cancer (NSCLC), one of the leading causes of cancer-related deaths worldwide. Unfortunately, 10–20 % of patients treated with radiotherapy experience radiation-induced lung injury (RILI) in the form of pneumonitis or fibrosis. The risk of these and other toxicities limits the radiation dose that can be delivered to the tumor and possibly hampers optimal tumor control [1], [2]. Compared to conventional radiation therapy using X-rays, proton therapy can reduce the occurrence and severity of these toxic side-effects. The depth-dose deposition characteristics of protons enable highly localized radiation delivery, allowing for better targeting of the tumor site while minimizing the dose to surrounding healthy tissues [3]. A promising approach to further reduce normal tissue damage is ‘FLASH’ radiotherapy. FLASH refers to the ultra-fast delivery of radiation at a dose rate exceeding 40 Gy/s, compared to the significantly lower dose rate of ≥ 0.01 Gy/s used in conventional radiotherapy [4]. Preclinical *in vitro* and *in vivo* studies have demonstrated improved normal tissue sparing with FLASH compared to conventional dose rates using electrons, photons, protons and carbon ions, while maintaining a similar tumor response- an observation known as the ‘FLASH-effect’ [5], [6], [7], [8], [9], [10], [11].

Several hypotheses have been proposed to explain the normal tissue sparing observed with FLASH, including the oxygen depletion theory, reduced DNA-damage and sparing of immune cells; however, the exact mechanism remains unclear [12], [13], [14]. Several recent mechanistic and modeling studies have been performed to elucidate the normal tissue-sparing effect of FLASH, particularly in relation to the oxygen depletion theory. This theory suggests that radiolytic oxygen depletion induces hypoxia, leading to increased radioresistance [15], [16], [17]. The advantages of FLASH are especially relevant for the treatment of lung cancer in patients with inflammatory lung diseases such as chronic obstructive pulmonary disease (COPD). Not only do these patients have a higher risk of lung cancer [18], [19], [20], but they are also more susceptible to RILI [21], [22], [23].

*In vitro* studies using cell cultures of primary lung cells from COPD patients could provide valuable insights into the effects and potential of FLASH in these patients. Culturing airway epithelial cells as organoids is increasingly recognized as a potential method for disease modelling, as well as studies on lung development, repair [24], [25] and radiobiology research [26], [27]. Organoids are defined as three-dimensional (3D) structures originating from stem or progenitor cells embedded in a matrix. Previous studies on salivary gland and intestinal organoids have demonstrated their potential for studying radiation responses and toxicity, making them a relevant alternative to animal experiments [28], [29].

The current study compared the effects of conventional (CONV) proton and proton FLASH on 3D organoid cultures of differentiated human primary bronchial epithelial cells (PBEC). The primary objective was to examine the functional consequences of these two modalities on airway epithelial cells using an organoid formation assay. This assay is a measure of the activity of lung progenitor cells that reside in the basal cell population of the pseudostratified airway epithelial cell layer. Lung progenitor cells can be viewed as local stem cells that can self-renew and differentiate into the various luminal epithelial cell types that constitute the airway epithelium. As a result, their function, as assessed in the organoid formation assay, is highly relevant when investigating the effects of FLASH. In addition, DNA damage was compared using γH2AX foci, a key marker for double-strand DNA breaks (DSBs). Their assessment can be used as an indicator of the extent of lethal DNA damage caused by radiation. Finally, bulk RNA sequencing (RNAseq) was performed to identify transcriptional differences between CONV and FLASH proton therapy in COPD patients.

## 2 Methods

### 3.1 Cell culture

Please refer to the supplemental methods for a more extensive description. Patient materials were obtained from the Leiden University Medical Center (LUMC) biobank, with approval from the local Medical Ethical Assessment Committee (METC). Cells were isolated from macroscopically normal lung tissue of patients undergoing surgical resection for lung cancer and stored as described elsewhere [30]. Cells from 6 COPD donors were thawed, expanded and cultured in drops of basement-membrane extract (BME) in 24-well plates for 2 weeks before the start of radiation.

### 3.3 Radiation

Irradiation took place at the Holland Proton Therapy Center (HollandPTC) in Delft, where a Varian ProBeam superconductive cyclotron supplies fixed horizontal proton beams ranging from 70 MeV to 250 MeV at various intensities, although ultra-high dose rate (UHDR) beams are only achievable at the maximum energy. The PBEC organoids were irradiated within a period of 10 weeks in batches of 2 donors. The organoids were transported from the LUMC at ∼ 37 °C in ambient air and placed in an incubator upon arrival at HPTC. Before irradiation, the medium was removed and the cell plates were positioned upright in front of a fixed horizontal proton beam.

**Fig. 1:**
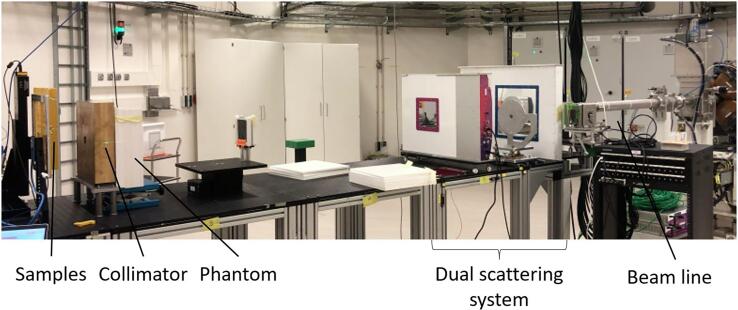
**Overview of beam-line setup**. From right to left: the beam line which delivers the protons followed by a dual scattering system. Next, the protons travel through the phantom and collimator to narrow the beam before it reaches the samples.

A 250 MeV pencil beam was laterally spread in the x-y direction using a single scattering foil to create a larger uniform field (Fig. 1), sufficient to cover a single well of a 24-well plate. The achieved field size was 18 mm with a uniformity of 97 %. The dose rate was defined as the total dose given uniformly to the field in a second (average dose rate) [31]. A total of 6 samples per condition per donor were positioned at the entrance of the Bragg peak and irradiated with 2 and 8 Gy at a dose rate of 15 Gy/min (CONV) or 40 Gy/s (FLASH). Control samples were handled similarly, but in a separate room to not expose them to any residual radiation.

### 3.4 Organoid formation assay

At 24 h and 7 days after radiation, organoids were dissociated and reseeded in BME hydrogel at 4000 cells/30 μL BME. After 10 days, brightfield microscope images (10x magnification) of the newly formed organoids were taken to assess organoid formation using open source OrganoSeg software [32].

### 3.5 Immunofluorescent staining, confocal microscopy, and analysis

At 1 h and 24 h after irradiation, organoids were fixed on a glass slide and stained with yH2AX antibody (Anti-phospho-Histone H2A.X [Ser139], 1:400, Sigma) and DAPI (1:300). Imaging of the slides was acquired with the Leica SP8 confocal microscope (63x, Leica Microsystems B.V., the Netherlands). For each condition, multiple organoids were imaged and ≥ 50 cells were analyzed. The number of yH2AX-foci were counted manually and 10 % of the images were counted by two independent researchers (intra class correlation coefficient 0.994). Cells were counted using an in-house created macro which was based on the FoCo macro from Lapytsko et al. [33]. Foci counts were expressed as the ratio of the number of foci to the number of cells.

### 3.7 RNA analysis

RNA was isolated seven days after radiation exposure. Samples of 3 randomly selected COPD donors were sent for bulk RNAseq to GenomeScan (Leiden, the Netherlands). A dataset of 15 samples was processed and the mapped read counts were subsequently analyzed using gene set enrichment analysis (GSEA) and the R2 Genomics Analysis and Visualization Platform database [34]. Relevant genes identified from the top differentially expressed genes (DEGs; see supplementary table 2 for top 25 DEGs) in the bulk RNA sequencing analysis (MDM2, DDB2, AEN, BAX, P21, GDF15, MKI67; primer sequences see supplementary table 1) were measured with qPCR in all 6 donors with reference genes ATP5b and RPL13a. Detailed description of the isolation, sequencing and analysis can be found in the supplemental methods.

### 3.8 Statistics

Data was analyzed using GraphPad Prism (v9.3.1) or SPSS statistics (v29.0, IBM). One-way ANOVA and Tukey test were used to assess differences. Outcomes are expressed as mean ± Standard Error of the Mean (SEM) unless stated otherwise. Differences at a P value < 0.05 were considered statistically significant.

## 3 Results

### 4.1 Baseline characteristics

In this study, cells from 6 donors were used to study the effects of CONV and FLASH proton radiation on PBEC organoids. The donors consisted of 4 males and 2 females. Their age ranged between 50 and 77 years (mean 63 years) and the mean BMI was 21 (range 18–23). There were 5 donors with COPD GOLD stage III and 1 donor with stage II; 3 donors were active smokers, 2 were former smokers and for 1 donor this was unknown.

### 4.2 γH2AX staining results

**Fig. 2:**
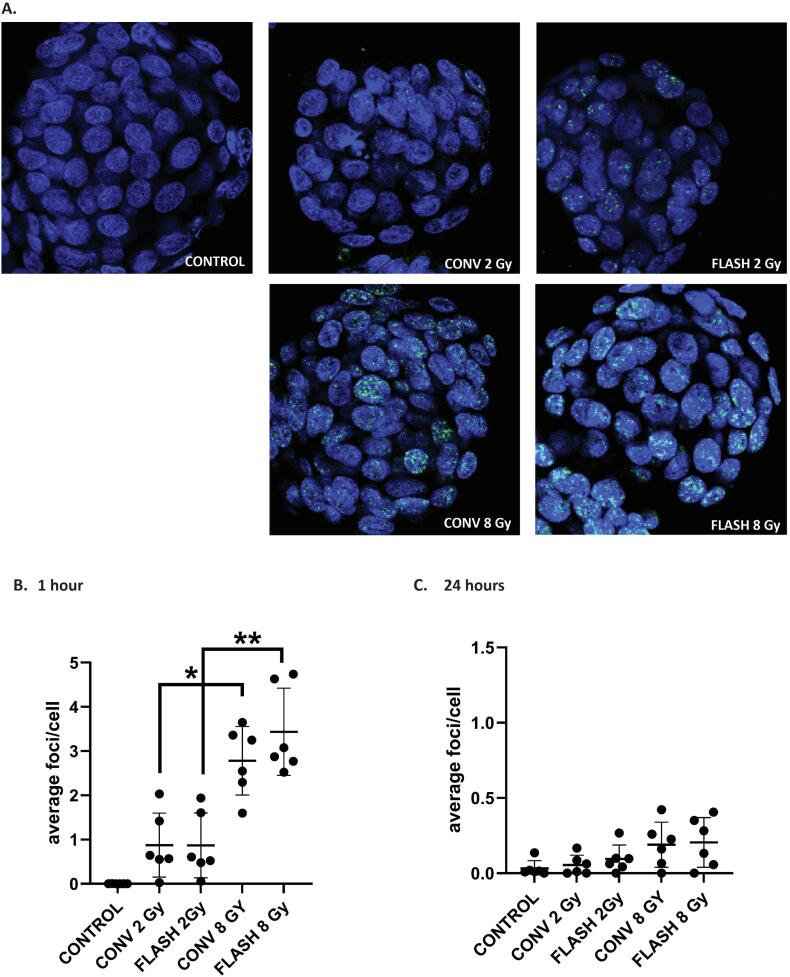
**γH2AX foci staining in PBEC organoids treated with 2 and 8 Gy CONV and FLASH proton radiation**. A) Representative images of γH2AX foci (green) per cell (DAPI, blue) in whole mounted organoids at 1 h following exposure to radiation (control, 2 and 8 Gy CONV and FLASH). B) Average number of γH2AX foci per cell at 1 h after radiation, 0.87 (2 Gy CONV), 2.78 (8 Gy CONV), 0.86 (2 Gy FLASH), 3.44 (8 Gy FLASH) and 0.001 foci/cell (CTRL), * = 0.0135, ** = 0.0064. C) Average number of γH2AX foci count per cell at 24 h after radiation, respectively 0.05, 0.19, 0.09, 0.2 and 0.03 foci/cell. (For interpretation of the references to colour in this figure legend, the reader is referred to the web version of this article.)

The yH2AX foci analysis revealed a significant dose-dependent increase of γH2AX foci per cell at 1 h for all donors between 2 and 8 Gy for both CONV (0.87 vs. 2.78 foci/cell, p = 0.0135) and FLASH (0.87 vs. 3.44 foci/cell, p = 0.0064; Fig. 2A, B). However, there was no significant difference between CONV and FLASH at both dose levels. After 24 h, γH2AX foci levels had normalized, albeit both 8 Gy treated samples seemed to have more residual foci compared to the 2 Gy treated samples and controls (Fig. 2C).

### 4.3 Organoid formation

**Fig. 3:**
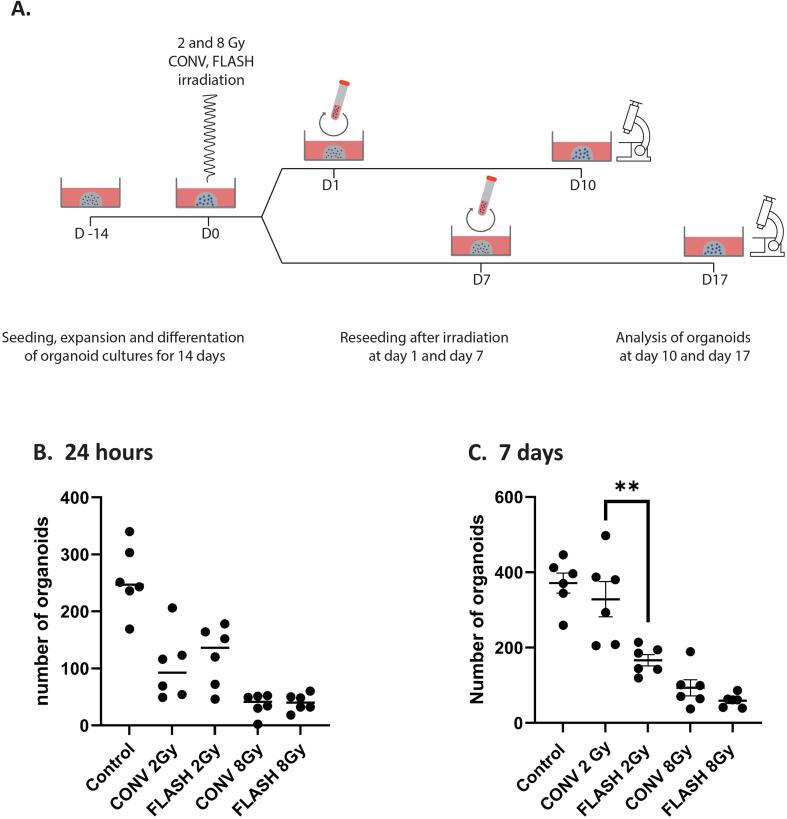
**Effect of CONV and FLASH proton radiation of PBEC organoids on organoid formation capacity.** Schematic overview of the experimental design (A) and the organoid counts (absolute number based on 15 images at 10x magnification per condition) in the organoid formation assay initiated at 24 h (D1; B) and 7 days (D7; C) after radiation.

Next, the effect of radiation on PBEC was assessed in an organoid formation assay (Fig. 3A). Analysis of organoids that expanded from reseeded irradiated organoids at 24 h showed a dose-dependent inhibition of organoid formation (Fig. 3B). At 7 days, 2 Gy FLASH treated organoids expanded significantly less than the CONV 2 Gy treated samples (respective value: 166 vs. 328 organoids; Fig. 3C, p = 0.008).

### 4.4 Transcriptomic analysis of FLASH and CONV irradiated samples

**Fig. 4:**
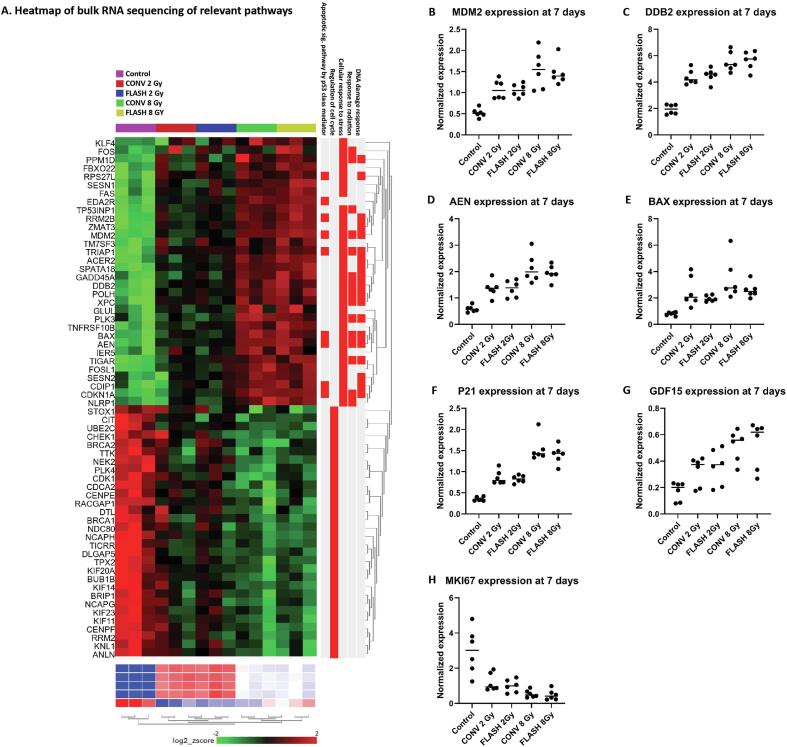
**Overview of transcriptomic analysis profile and gene expression.** (A) Heatmap of up- and downregulated genes (n = 3) for control, FLASH and CONV 2 and 8 Gy which shows differences in expression profiles between controls, 2 and 8 Gy samples, but no differences between FLASH and CONV; and (B-H) qPCR results (n = 6) of top differentially expressed genes (DEGs) selected from the bulk RNAseq analysis, respectively MDM2, DDB2, AEN, BAX, P21, GDF15 and MKI67.

Bulk RNAseq was performed on cultures from 3 donors treated with 2 and 8 Gy FLASH and CONV and controls to explore possible differences in the transcriptional response to both radiation modalities. However, the principal component analysis (PCA) of the RNAseq data showed more marked clustering based on donors than on treatment (supplemental figure 1). A comparison of gene expression between CONV and FLASH at both 2 Gy and 8 Gy did not reveal any differentially expressed genes (DEGs). Next, data from CONV and FLASH 8 Gy were compared to controls to identify DEGs between FLASH/CONV and non-irradiated samples. This resulted in a list of DEGs that were altered after both FLASH and CONV irradiation (top 25 DEGs, see supplemental table 2). Gene set enrichment analysis (GSEA) of the DEGs identified pathways related to the DNA damage response, response to radiation, response to stress and pathways related to apoptosis as key features of the transcriptomic profile. Irradiated samples showed a lower expression of genes related to regulation of the cell cycle compared to control samples (Fig. 4A). With the identification of key genes from the bulk RNAseq results, we performed qPCR analysis of the expression of these genes in all 6 donors. This showed a clear dose-dependent upregulation of MDM2, GDF15, DDB2, BAX, P21 and AEN expression following irradiation, although no differences between FLASH and CONV were noted. MKi67 expression, a marker of proliferation, was clearly decreased after irradiation and showed a dose-dependent trend (Fig. 4B-H).

### 4.5 Apoptotic marker staining

**Fig. 5:**
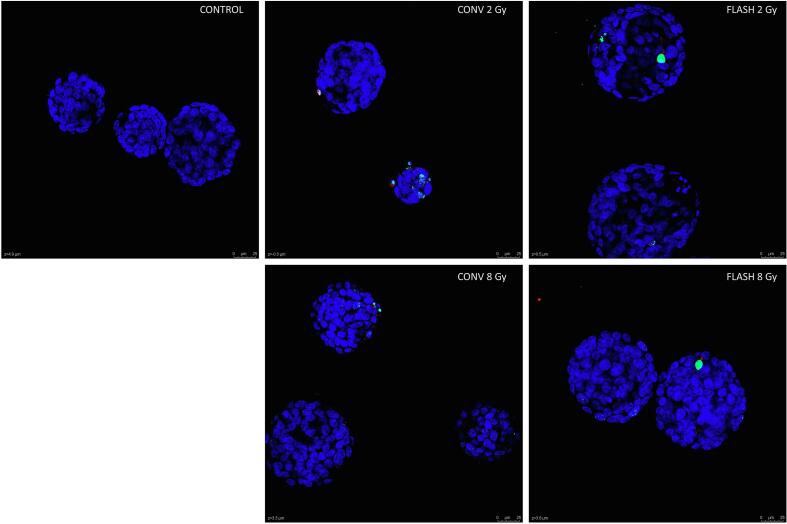
**Analysis of organoids from donor BR557.** Organoids stained for cleaved caspase-3 (apoptosis; red) and γH2AX (DNA breaks; green) and nuclei (blue), showing no clear differences between the irradiated samples at 24 h. (For interpretation of the references to colour in this figure legend, the reader is referred to the web version of this article.)

Because of the upregulation of expression of genes related to apoptosis pathways in the bulk RNAseq results, we checked the organoids at 24 h and 7 days after radiation for signs and differences of apoptosis using a combination of staining for cleaved caspase-3 and γH2AX. However, although staining for cleaved caspase-3 and γH2AX appeared more marked in the irradiated samples compared to controls, no clear differences were observed between CONV and FLASH (Fig. 5).

## 4 Discussion

This study investigated the effects of CONV and FLASH proton radiation on PBEC organoids derived from COPD patients. Proton radiation resulted in a dose-dependent increase in DNA damage markers, a reduction in organoid formation capacity, and alterations in the expression of various genes. However, no significant differences were observed between CONV and FLASH proton radiation, apart from a smaller decrease in organoid formation capacity in CONV-irradiated organoids compared to FLASH, assessed 7 days after 2 Gy radiation. This unexpected finding does not align with the anticipated sparing effect of FLASH.

To the best of our knowledge, this is the first study that investigated the effects of proton FLASH on patient-derived lung organoids, building on previous research that explored the effects of FLASH on primary human or animal lung cells and 3D cultures of other cell types. Fouillade et al. [35] reported that 4 Gy electron FLASH irradiation of primary bronchial epithelial cells cultured at the air–liquid interface (ALI-PBEC) resulted in a smaller decrease of p63^+^ expressing basal cells compared to conventional irradiation. Whereas this could indicate that FLASH spares basal cells *in vitro* in this specific culture system, functional effects on this cell population (that includes a progenitor population) were not evaluated. Klett at al. [28] investigated murine and human enteroids derived from the intestinal duodenum and found that enteroids treated with FLASH electrons (> 100 Gy/s) compared to CONV electrons (0.2 Gy/s) had greater colony forming potential and resembled proper intestinal polarity more frequently. A study using spheroids from the lung tumor cell line A549 by Khan et al. [36] did find a sparing effect related to the oxygen depletion theory [37], by showing that A549 spheroids with a hypoxic core had a 3-times higher clonogenic survival after electron FLASH irradiation compared to CONV. Combined, these studies show that *in vitro* studies can be used to demonstrate a positive effect of FLASH on specific target cells, i.e. sparing effect on progenitor cells, but it is still unclear why such a favorable effect of FLASH was not observed in our study. It could be speculated that 40 Gy/s might not be sufficient to achieve a sparing effect of FLASH. Other studies have shown that there could be a larger sparing effect when using a higher dose rate and that the threshold for the dose rate needed also depends on the dose sensitivity of the chosen endpoint, the tissue, and the reference CONV dose rate [38].

In our study, γH2AX foci analysis in both FLASH and CONV did not reveal differences between the types of radiation. Some previous studies have identified differences in DNA damage between FLASH and CONV, but others did not or only at extremely high doses. A study in IMR90 fetal lung fibroblasts observed less γH2AX foci for FLASH compared to CONV, however, this was observed at 20 Gy and a dose rate of 1000 Gy/s. Lower dose rates and lower dosages did not result in differences [39]. Tessonier et al. [40] investigated the lung cell lines A549 and H1347 with helium ions at a dose rate of 185 Gy/s versus 0.12 Gy/s and found no difference in γH2AX foci at 21 % oxygen, but did find less foci in the FLASH samples at 1 % oxygen. Kyle et al. [41] also only found a protective effect of FLASH in HCT-116 3D spheroids at 37 °C with 3 % oxygen using electrons at dose-rate of > 50 Gy/s. A closer examination of the type of DNA damage induced by FLASH compared to CONV revealed that FLASH reduces both complex DSBs and non-lethal single-strand breaks (SSBs) in plasmid DNA [42]. By contrast, other studies looking at plasmid DNA found that a proton beam at UHDR (48.6 Gy/s) showed less SSBs for FLASH treated samples, but no differences in DSBs induction. DSBs are considered to result in lethal DNA damage and a reduction is thought to contribute to the sparing effect of FLASH [43], [44]. These findings were obtained in a simple cell-free DNA model, but further investigation of SSBs in follow-up studies would be useful to assess their contribution to the normal tissue sparing effect of FLASH. The studies in cell lines and plasmid DNA also demonstrate that the effect of FLASH on γH2AX foci is highly dependent on dose rate, dose, and oxygen levels, highlighting the need for further in-depth research into the types of DNA damage induced [12].

Gene ontology analysis of our bulk RNAseq data on the effects of proton radiation revealed increased expression of genes related to the DNA damage response, apoptotic signaling pathway, response to radiation, cellular response to stress, and decreased expression of genes related to the cell cycle process. Results were confirmed by RT-PCR for a selected number of genes, and no differences between conventional and FLASH radiation were observed. Although the nature of these pathways in which differentially expressed genes were involved is not unexpected, to the best of our knowledge this is the first transcriptomic dataset of primary human lung cells after proton FLASH RT. Pathways that were notably absent in our dataset were pathways associated with inflammation and senescence, key features of the first response to radiation and RILI [1], [45]. Both have previously been found in transcriptomic analysis to be upregulated in lung endothelial cells [46], [47] and in mouse lungs [35] after radiation. Finally, because of the apoptotic gene expression, we performed additional staining for the apoptosis marker cleaved caspase-3 in combination with γH2AX, which showed a limited increase in apoptosis in the irradiated samples, but no differences between the dosages or treatment modalities, which is in line with results from the transcriptomic analysis.

This study has four main limitations. First, the organoids consisted of only PBEC, and although these cells form a heterogeneous population of basal cells, ciliated cells and secretory cells and other more rare epithelial subsets, an interplay with immune cells, endothelial cells and/or fibroblasts might be essential to capture more aspects of radiation toxicity in the lung, as was recently demonstrated by Dasgupta et al [48]. Second, the cells were irradiated in an upright position, without medium and at room temperature with ambient air. Temperature and oxygen levels may play a crucial role in the FLASH effect as was described before, although there are also *in vitro* studies that did not include any alterations in oxygen levels and temperature and nevertheless observed normal tissue sparing with FLASH [28], [35], [39]. Third, we used a larger field size derived from a proton pencil beam similar to other proton FLASH studies [49], [50], [51]. However, we did not consider the effects of using pencil beam scanning, rescanning or other radiation delivery characteristics (e.g., time structure of the beam, fractionation, beam pauses), all of which are known to potentially influence the magnitude of the sparing effect of FLASH. These aspects are highly relevant for future clinical practice, as scanning delivery is often required to achieve a sufficiently large field size for tumor treatment, and fractionation is a standard approach [8]. Finally, we used cells from patients with moderate COPD, because we aimed to explore the effects of FLASH in cells from patients at increased risk of symptomatic RILI and evaluate if FLASH could be a strategy to reduce the risk of toxicity in these patients. However, cells from COPD patients could react differently to radiation compared to those from healthy controls [52], and therefore we cannot exclude the possibility that the absence of a beneficial effect of FLASH is due to the use of COPD cells. At the same time, since these types of patients more frequently develop lung cancer, the use of primary COPD cells from multiple donors is also a strength of this study and improves the translational value of our results. Moreover, organoid models are quickly gaining importance in radiobiology research [53] and culturing in hydrogel adds to the translational value since it is less stiff than the tissue culture plastic of a traditional submerged culture or the (PET) membrane of an air–liquid interface model. The main readout, progenitor function, can be considered a highly relevant and functional readout, essential for the repopulation of the healthy cell population and significant for the development of pulmonary fibrosis [54], [55]. Finally, another strength of our study is that we performed an unbiased transcriptomic analysis with the use of bulk RNAseq and identified gene sets that are of importance for future research of the normal lung tissue radiation response. Therefore, this data can be used to design new *in vitro* experiments with FLASH.

This study is the first to examine the effects of CONV and proton FLASH on primary human bronchial epithelial organoids *in vitro,* derived from a highly relevant patient group (COPD). The bulk RNAseq has identified pathways that are relevant for radiation research in the lung, but differences between FLASH and CONV were not observed. The lack of a sparing effect with FLASH in our study, indicates that more advanced models may be needed, including multiple lung /cell types (immune, epithelial, endothelial, and/or mesenchymal cells) to mimic the microenvironment with its network of intercellular communication. Furthermore, it may be relevant to vary oxygen levels, temperature, include mechanical force (airflow, stretch and compression), focus on alveolar epithelial cells, and use cells from various patient groups. Defining the conditions needed to induce the FLASH sparing effect *in vitro* could pave the way for a patient-specific approach, relevant to the designated patient group without the need for animal models.

## Conclusion

Using primary bronchial epithelial organoids, this study shows that proton radiation decreases organoid formation capacity and modulates expression of a variety of genes, but no substantial differences between CONV and FLASH radiation modalities were observed. This study adds to the few studies that have investigated the effects of FLASH *in vitro.* It contributes to a further understanding of the FLASH sparing effect, and the development of adequate *in vitro* human lung models that can be used to study the effects of FLASH. Collectively, this may pave the way for treating lung cancer patients with proton FLASH.

## CRediT authorship contribution statement

**Merian E. Kuipers:** Formal analysis, Funding acquisition, Visualization, Writing – original draft. **Floriane van Liefferinge:** Formal analysis, Investigation, Visualization. **Ernst van der Wal:** Investigation, Resources. **Marta Rovituso:** Investigation, Resources, Writing – review & editing. **Annelies M. Slats:** Supervision. **Pieter S. Hiemstra:** Supervision, Writing – review & editing. **Krista C.J. Van Doorn-Wink:** Conceptualization, Supervision, Writing – review & editing.

## Funding

This project was supported by the Wassink-Hesp Foundation.

Declaration of Generative AI and AI-assisted technologies in the writing process.

During the preparation of this work the authors used ChatGTP in order to optimize, shorten and check the abstract of this manuscript. Additionally, the authors utilized ChatGTP to refine English grammar and enhance readibility. After using this tool, the authors reviewed and edited the content as needed and take full responsibility for the content of the publication.

## Declaration of Competing Interest

The authors declare the following financial interests/personal relationships which may be considered as potential competing interests: Merian Kuipers reports financial support was provided by the Wassink-Hesp Foundation. The other authors declare that they have no known competing financial interests or personal relationships that could have appeared to influence the work reported in this paper.
