# Supplementary material for: Effect of FLASH proton therapy on primary bronchial epithelial cell organoids
Source: Clin Transl Radiat Oncol. 2025 Jan 29;52:100927. doi: 10.1016/j.ctro.2025.100927 (PMC11833640; doi:10.1016/j.ctro.2025.100927)

**Supplemental methods**

3.1 Cell culture

All patient cells were retrieved from our biobank which has been approved by the local Medical Ethical Assessment Committee (METC). Cells were isolated from macroscopically normal lung tissue obtained from patients undergoing resection surgery for lung cancer at the Leiden University Medical Center, the Netherlands. The lung tissue that was derived was enrolled in the biobank via a no-objection system for coded anonymous further use of such tissue ([www.coreon.org](http://www.coreon.org/)). However, since 01-09-2021, patients are enrolled in the biobank using active informed consent in accordance with local regulations from the LUMC biobank with approval by the institutional medical ethical committee (B20.042/Ab/ab and B20.042/Kb/kb). Approval documents are available upon request.

3.2 Cell culture

Cells from our biobank from COPD patients were thawed from liquid nitrogen-stored cryovials and seeded in a T75 flask coated with 30 μg/mL bovine type-1 collagen (PureCol®, Advanced BioMatrix), 10 μg/mL fibronectin (Promocell, PromoKine, Bio-connect) and 10 μg/mL BSA (ThermoFisher Scientific) in BEpiCM-b basal medium (ScienCell Research Laboratories, Sanbio), supplemented with 100 U/mL penicillin (Lonza), 100 μg/mL streptomycin (Lonza) and bronchial epithelial cell growth supplement (ScienCell). After expansion, cells were trypsinized in 0.03% (w/v) trypsin (ThermoFisher Scientific), 0.01% (w/v) EDTA (BDH, Poole, UK), 0.1% glucose (BDH) in phosphate buffered saline (PBS). Next, cells were seeded in drops of 30-60 μL basement-membrane extract (BME; Cultrex PathClear Reduced Growth Factor BME, BioTechne) in 24-well plates with 4000-8000 cells/droplet and cultured in airway organoid medium (PMID: 30643021 (1)). After two weeks of culture, organoids had expanded and were transported to the proton therapy facility at approximately 37°C in ambient air and immediately placed in an incubator upon arrival. Cells were incubated at 37°C with 5% CO2, airway organoid medium (PMID: 30643021) was refreshed twice a week.

3.3 Radiation

The irradiation of the cells was conducted at the Holland Proton Therapy Center (HollandPTC) in Delft. Immediately before irradiation, the medium was removed, and the cell plates were positioned upright in front of the proton beam line. The latter was a fixed horizontal beam equipped with a passive scattering system that creates large and uniform irradiation fields (2). A Varian ProBeam superconductive cyclotron supplies proton beams ranging from 70 MeV to 250 MeV at various intensities, athough Ultra high dose rate (UHDR) beams are only achievable at the maximum energy.

The 250 MeV pencil beam was laterally spread in the x-y direction using a single scattering foil to create a larger uniform field area, sufficient to cover a single well of a 24-well plate. The achieved field size was 18 mm with a uniformity of 97%. The samples were positioned at the entrance of the Bragg peak, at a depth of 10 cm in RW3. At this depth, two dose rates were calibrated for irradiation: a conventional dose rate at 15 Gy/min and an UHDR at 40 Gy/sec. Doses of 2 and 8 Gy were delivered, corresponding to irradiation times of approximately 50 msec and 200 msec, respectively, for UHDR.

Samples were placed in four different wells per plate, spaced a few centimeters apart. A total of 4 well-plates (16 samples per run) were positioned on a motorized linear stage with preset coordinates. After each run, the position was switched remotely, allowing the next irradiation to be performed. Following a waiting time of about 15-20 minutes, the plates were exchanged for the next set of irradiations. Control samples were treated simultaneously by removing the medium and exposing them to room temperature. They were kept in a separate room as to not expose them to any residual radiation.

For dosimetry and field characterization, an Advanced Markus chamber was used together with an ionization chamber built for UHDR (FlashQ). The cross-calibration between the advanced Markus chamber and the FlashQ ensured accurate and controlled irradiations.

3.4 Organoid formation assay

24 hours after radiation, BME droplets with organoids were carefully dissociated with ice-cold PBS and transferred to a 15 ml tube. Organoids were centrifuged for 5 minutes at 1200rpm at 4°C. Next, the supernatant was aspirated and 400μL TrypLE (Gibco, USA) was added to the cell pellet, incubated at 37° and thoroughly pipetted to obtain a single cell suspension. Trypsinization was stopped by adding 800μL of organoid base medium. Next, cells were centrifuged 5 minutes at 1200rpm and then seeded in BME at a concentration of 4000 cells per 30μL BME. Three droplets were seeded per condition. After 10 days of culture, brightfield microscope images (10x magnification) of the newly formed organoids were taken for analysis. Five pictures per droplet were taken for each condition to create an accurate representation of the organoids formed. In general, the plane with the most organoids visible was sought when acquiring the image. Analysis was performed as described by Borten et al. in MATLAB (version R2022b) using OrganoSeg, an open-source software that allows semi-automatic quantification of organoids (PMID: 29593296 (3)).

3.5 Immunofluorescent staining

At 1 hour and 24 hours after irradiation, organoids were fixed for immunofluorescent staining. The droplets were carefully disrupted with ice-cold PBS and shaken on ice for 15 minutes to liquify the BME. The whole organoids suspension was then centrifuged for 6 min at 450 rpm onto a glass slide. Cells were fixed with 4% paraformaldehyde (PFA; Merck, Germany) for 15 minutes. Before staining, cells were permeabilized with ice-cold methanol for 10 minutes. Slides were washed with PBS and then incubated overnight with 50μL yH2AX antibody (Anti-phospho-Histone H2A.X [Ser139] Alexa-Fluor 488 conjugated, monoclonal mouse IgG1 antibody, Sigma; dilution 1:400) in PBS containing 5% BSA and 0.3% Triton-X100 in PBS (PBT). After washing with PBS, 50μL of DAPI in PBT (1:300) was added for 30 minutes. Cells were washed again with PBS and mounted with prolong gold anti-fading reagent (ThermoScientific) and a coverslip.

3.6 Confocal microscopy and analysis

Imaging of the slides was acquired with the Leica SP8 confocal microscope (Leica Microsystems B.V., the Netherlands), magnification 63x, and the LAS AF lite software (Leica Microsystems B.V., the Netherlands). For each condition, multiple organoids were imaged, and a z-stack was acquired. A representative image from each condition was randomly selected for analysis and at least 50 cells per condition were analysed. Analysis was done by manually counting the number of yH2AX-foci. To allow accurate representation of the counts, 10% of the images were counted by two independent researchers which showed good agreement (intra class correlation coefficient 0.994). The number of cells was counted using an in-house created macro which was based on the FoCo macro from Lapytsko et al. (PMID: 26589438 (4)). The number of foci is represented by dividing the number of foci by the number of cells.

3.7 RNA Isolation and bulk RNA sequencing

Seven days after radiation exposure, droplets were disrupted with ice-cold PBS and transferred to a 15ml tube. After 5 minutes of centrifugation at 1200rpm at 4°C, the supernatant was aspirated and organoids were lysed with 200μL RNA lysis buffer (Promega, USA) and stored at -20°C. During processing, the RNA was isolated using the Maxwell 16 LEV simplyRNA Tissue Kit (Promega, USA) according to the manufacturer’s protocol. Bulk RNA sequencing (RNA-seq) was performed on samples from three COPD donors, which were sent to GenomeScan in Leiden, the Netherlands. At GenomeScan, mRNA was fragmented, followed by cDNA synthesis for the subsequent ligation of sequencing adapters and PCR amplification. The quality and yield of the prepared samples were assessed using a fragment analyzer, showing the expected fragment size distribution between 300 and 500 base pairs. The quality of the library preparation was further confirmed by mapping the raw sequencing data to annotated genomic references. Sequencing was conducted on the NovaSeq6000 platform following the manufacturer's protocol, with an input concentration of 1.1 nM DNA. The Illumina data analysis pipeline, using RTA version 2.4.11 and Bcl2fastq version 17, handled image analysis, base calling, and quality control checks. Trimmed sequence reads, generated by removing potential adapter sequences with cutadapt v2.10, were mapped to the Homo sapiens.GRCh37.75 reference genome using the Tophat aligner (v2.0.14), which employs the Burrows-Wheeler Transform for short read alignment. Read frequencies were computed as read counts, and differential expression analysis was conducted using the DESeq2 software package. The analysis compared groups: 2 Gy FLASH vs. 2 Gy CONV, 8 Gy FLASH vs. 8 Gy CONV, and both 8 Gy FLASH and 8 Gy CONV against the control group. Statistically significant differences were observed only in the comparison between the treated and control samples. Genes with a log2 fold change greater than 0.5 and an adjusted P-value (q-value) of less than 0.05 were deemed significant. DESeq2 applied the Benjamini-Hochberg method to adjust for false discovery rates (FDR).

3.8 Genes set analysis

The gene sets were identified with a gene set enrichment analysis (GSEA) using the online G-profiler platform, accessed via the website <https://biit.cs.ut.ee/gprofiler/gost.> The positively and negatively upregulated DEGs with a q value <0.05 were uploaded separately in the investigate gene sets column of the website, and the overlapping gene sets were determined. The gene sets identified were compared to literature to determine the most relevant sets. Gene sets were plotted in a heat map using the R2 Genomics Analysis and Visualization Platform database (REF).

3.9 cDNA synthesis and qPCR

For qPCR analysis, cDNA synthesis was performed using a mix of Oligo(dT)15 (Qiagen, Germany), dNTP (Promega, USA), M-MLV reverse transcriptase (Promega, USA) and RNAsin (Promega, USA). For qPCR analysis, IQ SYBR green Supermix (Bio-Rad, the Netherlands) and primers were added to the diluted cDNA. Out of the top 25 negatively and positively upregulated DEGs (see supplemental table 1), 7 genes were chosen based on comparison with literature and previous experience (see supplemental table 2). For all 6 donors, these relevant genes were measured in triplicates with the Real-Time PCR Detection System CFX96 (Bio-Rad, the Netherlands). Expression of the reference genes ATP5b and RPL13a was used to calculate the normalized RNA levels. All calculations were done using the Bio-Rad CFX manager 4.0 software (Bio-Rad, the Netherlands).

3.10 Statistics

Data was analysed using GraphPad Prism (version 9.3.1) or SPSS statistics (version 29.0, IBM). One-way ANOVA and Tukey test were performed to determine overall differences between groups and singular differences, respectively. Outcomes are expressed as mean ± Standard Error of the Mean (SEM) unless stated otherwise. Differences at P value <0.05 were considered statistically significant.

**References**

1. Sachs N, Papaspyropoulos A, Zomer-van Ommen DD, Heo I, Bottinger L, Klay D, et al. Long-term expanding human airway organoids for disease modeling. Embo j. 2019;38(4).

2. M. Rovituso CFG, E. van der Wal, W. van Burik, A. Ibrahimi, H. Rituerto Prieto, J. M. C. Brown, U. Weber, Y. Simeonov, M. Fontana, D. Lathouwers, M. van Vulpen, M. Hoogeman. Characterisation of the HollandPTC R&D proton beamline for physics and radiobiology studies. arXiv. 2023.

3. Borten MA, Bajikar SS, Sasaki N, Clevers H, Janes KA. Automated brightfield morphometry of 3D organoid populations by OrganoSeg. Sci Rep. 2018;8(1):5319.

4. Lapytsko A, Kollarovic G, Ivanova L, Studencka M, Schaber J. FoCo: a simple and robust quantification algorithm of nuclear foci. BMC Bioinformatics. 162015.

**Supplemental table 1.** Overview of primer sequences

| **Gene name** | **forward sequence (5' to 3')** | **reverse sequence (5' to 3')** |
| --- | --- | --- |
| AEN | CATCGCTGACTACCGTACCC | TGCTGCTGTCAGGTTCTCTG |
| BAX | AGCTGAGCGAGTGTCTCAAG | GAGAGGAGGCCGTCCCA |
| DDB2 | GAGCATCACTGGGCTGAAGT | CGGATCTCGCTCTTCTGGTC |
| GDF15 | GGTGAATGGCTCTCAGATG | CACTTCTGGCGTGAGTATC |
| MDM2 | CCGGATCTTGATGCTGGTGT | ATCACTCTCCCCTGCCTGAT |
| MKi67 | AGACCTCCCCAGAGATGGAC | TGCACACCTCTTGACACTCC |
| P21/CDKNA1 | TTGTCACCGAGACACCACTG | AGTGGTAGAAATCTGTCATGCT |
| ATP5B1B | TCACCCAGGCTGGTTCAGA | AGTGGCCAGGGTAGGCTGAT |
| RPL13A | AAGGTGGTGGTCGTACGCTGTG | CGGGAAGGGTTGGTGTTCATCC |

**Supplemental table 2.** List of positively and negatively top 25 upregulated differentially expressed genes

| **Top 25 negatively upregulated genes in control versus 8 Gy CONV and FLASH** | | | | |
| --- | --- | --- | --- | --- |
| **Ensembl ID** | **GeneName** | **log2FoldChange(log2(Control_HPTC)-log2(8_Gy))** | **P-Value** | **P-Adjusted (q-value)** |
| ENSG00000251095 |  | -2,113 | 1,06818E-35 | 1,86952E-31 |
| ENSG00000124762 | CDKN1A | -1,652 | 1,23388E-31 | 1,07977E-27 |
| ENSG00000144452 | ABCA12 | -2,086 | 2,55003E-31 | 1,48769E-27 |
| ENSG00000135679 | MDM2 | -1,348 | 7,13879E-31 | 3,12358E-27 |
| ENSG00000172667 | ZMAT3 | -1,373 | 8,93928E-25 | 3,12911E-21 |
| ENSG00000112659 | CUL9 | -1,332 | 3,36192E-20 | 1,23692E-16 |
| ENSG00000168918 | INPP5D | -1,668 | 5,37001E-19 | 1,64645E-15 |
| ENSG00000228526 | MIR34AHG | -1,311 | 1,32962E-18 | 2,90887E-15 |
| ENSG00000131080 | EDA2R | -1,472 | 4,47439E-18 | 8,7012E-15 |
| ENSG00000223883 | LINC01707 | -1,941 | 8,19957E-18 | 2,04872E-14 |
| ENSG00000205420 | KRT6A | -1,531 | 1,6041E-16 | 2,95091E-13 |
| ENSG00000182601 | HS3ST4 | -1,719 | 8,87391E-16 | 1,36037E-12 |
| ENSG00000173535 | TNFRSF10C | -1,630 | 7,83657E-15 | 1,02973E-11 |
| ENSG00000078237 | TIGAR | -1,305 | 1,67746E-14 | 1,83493E-11 |
| ENSG00000048392 | RRM2B | -1,123 | 2,69553E-14 | 2,62095E-11 |
| ENSG00000163071 | SPATA18 | -1,080 | 6,36892E-14 | 5,57344E-11 |
| ENSG00000181026 | AEN | -1,298 | 1,04464E-13 | 1,28115E-10 |
| ENSG00000285882 |  | -1,533 | 1,38781E-13 | 1,59563E-10 |
| ENSG00000087088 | BAX | -1,273 | 6,23104E-13 | 6,74272E-10 |
| ENSG00000120889 | TNFRSF10B | -1,027 | 9,29518E-13 | 6,96006E-10 |
| ENSG00000176912 | TYMSOS | -1,591 | 9,54414E-13 | 6,96006E-10 |
| ENSG00000130513 | GDF15 | -1,392 | 2,52454E-12 | 2,32207E-09 |
| ENSG00000175592 | FOSL1 | -1,649 | 8,3496E-12 | 6,67822E-09 |
| ENSG00000134574 | DDB2 | -1,048 | 1,33882E-11 | 1,02621E-08 |
| **Top 25 positively upregulated genes in control versus 8 Gy CONV and FLASH** | | | | |
| **Ensembl ID** | **GeneName** | **log2FoldChange(log2(Control_HPTC)-log2(8_Gy))** | **P-Value** | **P-Adjusted (q-value)** |
| ENSG00000117724 | CENPF | 1,682 | 1,95263E-15 | 2,62885E-12 |
| ENSG00000117507 | FMO6P | 1,292 | 2,46978E-15 | 3,08758E-12 |
| ENSG00000148773 | MKI67 | 1,662 | 3,5122E-14 | 3,23529E-11 |
| ENSG00000064692 | SNCAIP | 1,554 | 1,877E-13 | 1,56434E-10 |
| ENSG00000131747 | TOP2A | 1,501 | 5,56884E-13 | 4,43027E-10 |
| ENSG00000024526 | DEPDC1 | 1,664414743 | 2,93842E-12 | 2,57406E-09 |
| ENSG00000088325 | TPX2 | 1,454 | 1,07021E-11 | 7,20414E-09 |
| ENSG00000140479 | PCSK6 | 1,449 | 1,32319E-11 | 8,57723E-09 |
| ENSG00000170312 | CDK1 | 1,459038553 | 6,76092E-11 | 4,60644E-08 |
| ENSG00000138160 | KIF11 | 1,245923582 | 2,44115E-09 | 1,21371E-06 |
| ENSG00000011426 | ANLN | 1,373336691 | 2,63172E-09 | 1,27403E-06 |
| ENSG00000142731 | PLK4 | 1,244045185 | 2,7277E-09 | 1,28663E-06 |
| ENSG00000138778 | CENPE | 1,367002284 | 6,25855E-09 | 2,8081E-06 |
| ENSG00000169679 | BUB1 | 1,35995491 | 7,19581E-09 | 3,15177E-06 |
| ENSG00000254951 |  | 1,390508407 | 8,95126E-09 | 3,82947E-06 |
| ENSG00000109805 | NCAPG | 1,385555812 | 9,26392E-09 | 3,87316E-06 |
| ENSG00000114204 | SERPINI2 | 1,324 | 1,0243E-08 | 3,84214E-06 |
| ENSG00000079308 | TNS1 | 1,175602293 | 1,89298E-08 | 7,25484E-06 |
| ENSG00000286522 | H3C2 | 1,294 | 2,23788E-08 | 7,99334E-06 |
| ENSG00000184357 | H1-5 | 1,281 | 2,83676E-08 | 9,92979E-06 |
| ENSG00000183856 | IQGAP3 | 1,350901034 | 2,92999E-08 | 1,03654E-05 |
| ENSG00000066279 | ASPM | 1,275 | 3,53284E-08 | 1,16664E-05 |
| ENSG00000137807 | KIF23 | 1,275 | 4,34423E-08 | 1,40801E-05 |
| ENSG00000118193 | KIF14 | 1,327495216 | 5,06039E-08 | 1,66234E-05 |

**Supplemental figure 1**. Principal component analysis (PCA) plots of A. samples grouped by individual donors and B. samples grouped by treatment modality.


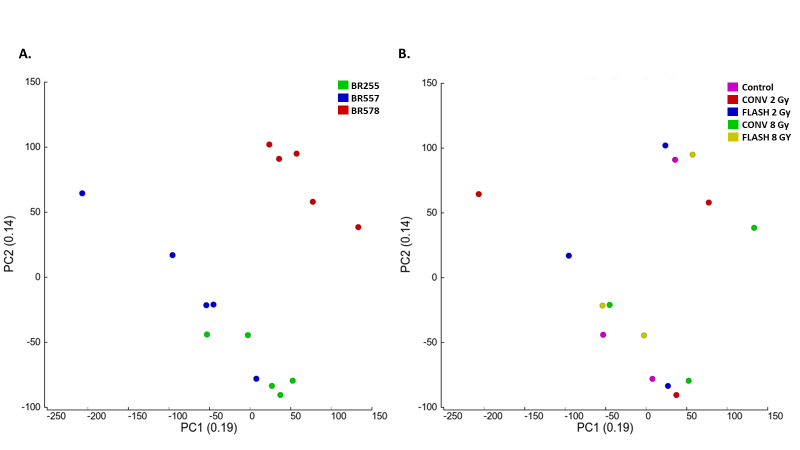

Supplement: Supplementary Data 1 [file mmc1.docx]
